# A case of native lung hyperinflation after single lung transplantation treated with lung volume reduction coils

**DOI:** 10.1016/j.jhlto.2024.100103

**Published:** 2024-05-09

**Authors:** Michael Perch, Kristine Jensen, Anna Kalhauge, Henrik-Jessen Hansen, Jann Mortensen

**Affiliations:** a Department of Cardiology, Section for Lung Transplantation, Rigshospitalet, Copenhagen, Denmark; b Department of Clinical Medicine, Copenhagen University Hospital, Rigshospitalet, Copenhagen, Denmark; c Department of Radiology, Rigshospitalet, Copenhagen, Denmark; d Department of Cardiovascular and Thoracic Surgery, Rigshospitalet, Copenhagen, Denmark; e Department of Clinical Physiology and Nuclear Medicine, Rigshospitalet, Copenhagen, Denmark

**Keywords:** lung transplantation, hyperinflation, lung volume reduction, coil, emphysema

## Abstract

Single lung transplantation (SLTx) has been used to treat end-stage lung disease. A common complication after SLTx is hyperinflation of the native emphysematous lung (NLH). Previous attempts to reduce the NLH have been tried using surgery, endoscopic valves, etc. with variable outcomes. Lung volume reduction coils can be used for treating hyperinflation in emphysema patients irrespective of collateral ventilation (CV). We here report a case of native lung hyperinflation in SLTx treated with lung volume reduction coils. This treatment appears to be a safe treatment option when dealing with CV-positive NLH in SLTx recipients.

Single lung transplantation (SLTx) has been used to treat end-stage lung disease. A common complication after SLTx is hyperinflation of the native emphysematous lung (NLH) which is defined by radiologic expansion of the native lung leading to compression of the diaphragm, changed respiratory dynamics, and eventually a mediastinal shift compressing the graft and reducing the return blood flow to the heart, which all lead to increased symptoms of breathlessness.^1^ Reliving the graft by reducing the volume of the hyperinflated native lung can be done since there typically is only little or no ventilatory function left in the native lung. This has been done using surgical techniques such as pneumonectomy, lobectomy, or even prophylactic lung volume reduction surgery at the time of transplant.^2^ Unfortunately results have varied, and morbidity and mortality have been high. The first minimally invasive approach to NLH was reported in 2007 using endobronchial valves as compassionate use in an SLTx case.^3^ Additional smaller case series have shown improvement in some recipients. Since then the presence of collateral ventilation (CV) has been identified as important for outcome when treating hyperinflation with valves in emphysema patients.^4^ CV is a consequence of incomplete fissures between the lung lobes and is often present in patients with NLH, generally making valves unsuitable for treating NLH. Lung volume reduction coils (LVRC) have been introduced for treating hyperinflation in emphysema, including patients who are CV-positive.^5^

We here present a case of NLH after SLTx treated with LVRC. The case is a middle-aged female with normal antitrypsin, who received a left SLTx due to severe emphysema. The transplant procedure was uneventful and had a good clinical outcome. She improved her quality of life for several years, although over time she continued to decline in forced expiratory volume in 1 second (FEV1) and forced vital capacity (FVC) (Figure 1), with increasing symptoms of dyspnea and intermittent right-sided chest pain. By spring 2017 her condition had deteriorated, and radiologic images showed increasing hyperinflation of the native lung as seen in Figure 2 which was confirmed on computer tomography with compression of the mediastinum as seen in Figure 3. The lobar fissures were incomplete and therefore, assessed as CV positive. Body plethysmography did confirm severe hyperinflation with increased total lung capacity (TLC) of 5.26 liter equivalent to 107% and increased residual volume (RV) of 4.08 equivalent to 242% with an RV/TLC ratio of 78% before treatment. Evaluating the magnitude of native lung hyperinflation (NLH) in relation to graft dysfunction, or their synergistic effect, presents a clinical conundrum. Optimal assessment entails discerning compelling evidence of hyperinflation in the native lung, exerting pressure on the graft, while diligently excluding alternative etiologies of graft dysfunction. In our case, graft dysfunction was evaluated through a comprehensive approach combining imaging and bronchoalveolar lavage (BAL). Radiological examinations revealed no indications of acute graft dysfunction or infection, while microbiological analysis of BAL fluid yielded negative results. The cellular composition of the BAL fluid was characterized by macrophages (71%), neutrophils (21%), and lymphocytes (8%). Consequently, we attributed the patient's deterioration primarily to native lung hyperinflation (NLH).

**Figure 1:**
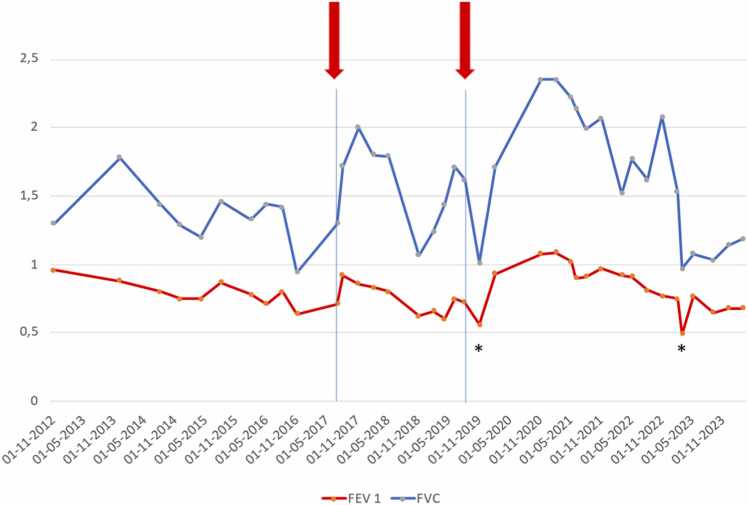
Trajectory of FEV1 and FVC until most recent spirometry. Red arrows symbolize LVRC treatments. *Drop in FEV1 and FVC due to infection. FEV1, forced expiratory volume in 1 second; FVC, forced vital capacity; LVRC, lung volume reduction coils.

**Figure 2:**
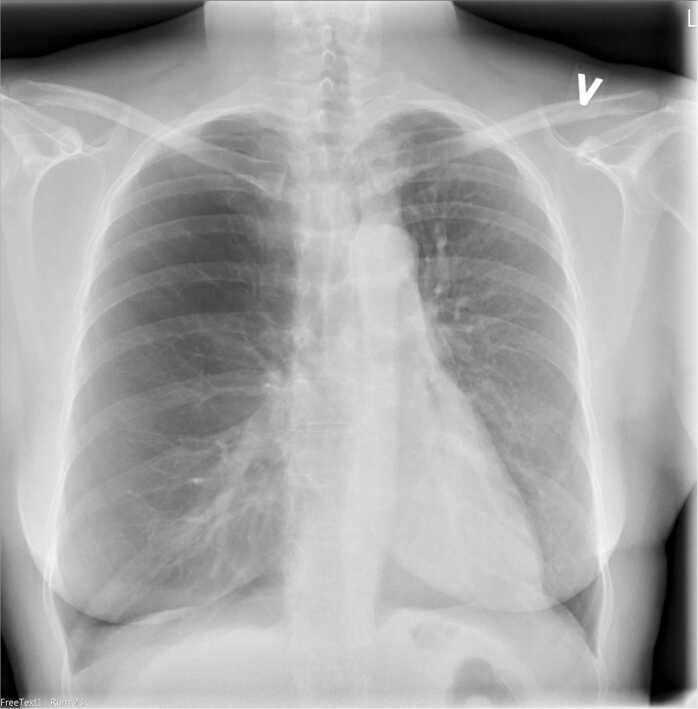
Chest X-ray before LVRC treatment. LVRC, lung volume reduction coils.

**Figure 3:**
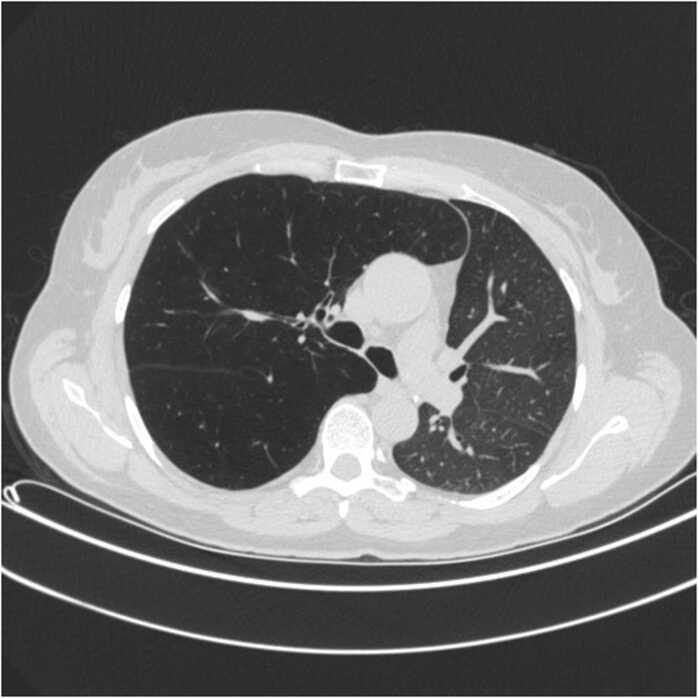
Preoperative chest CT depicting the transplanted left lung and the hyperinflated right native lung compressing the mediastinum toward the left. CT, computer tomography.

LVRCs are placed through a bronchoscope under the guidance of fluorescens in general anesthesia. As many LVRCs as anatomically possible were implanted in separate segments. A total of 12 LVRCs (3 × 100 mm, 4 × 125 mm, and 5 × 150 mm) were placed in the upper and middle lobe of the native lung as seen in Figure 4 under prophylactic prednisone and oral antibiotics as suggested by the manufacturer. The procedure lasted a total of 58 minutes and was without complications. The patient was discharged after 5 days. Three months later she had less dyspnea and had improved modified Medical Research Council by 1. Both FEV1 and FVC had improved as seen in Figure 1 and RV/TLC ratio had declined as seen in Figure 5. Six-minute walking distance had increased by 19 m. She did no longer experience any chest pain.

**Figure 4:**
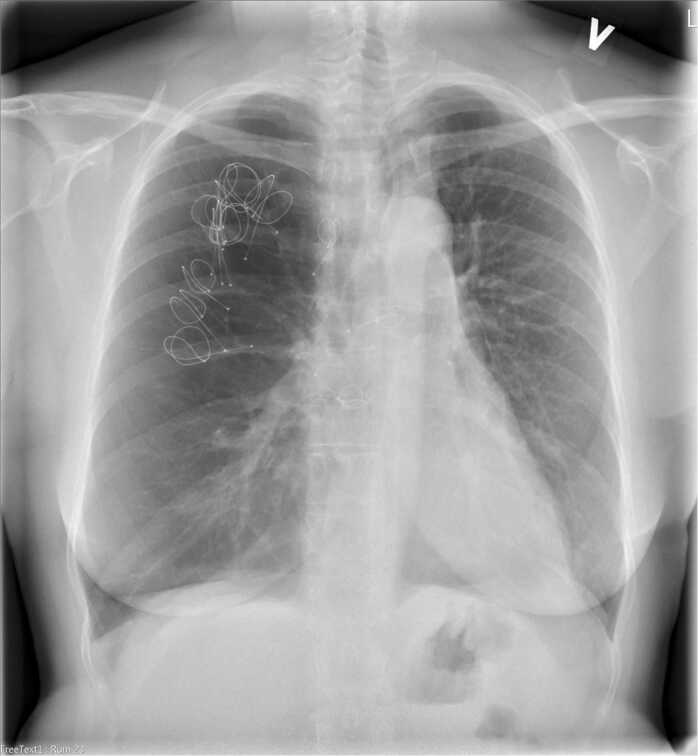
Postoperative chest X-ray after first LVRC treatment of right upper and middle lobes. LVRC, lung volume reduction coils.

**Figure 5:**
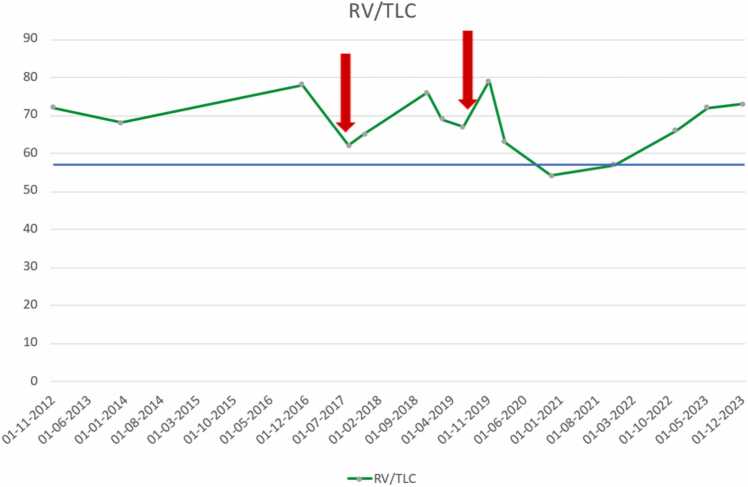
Trajectory of RV/TLC ratio over time. The blue line depicts RV/TLC = 58%, When above 58 is equivalent to hyperinflation. RV/TLC, residual volume/total lung capacity.

Two years after the initial treatment, symptoms reoccurred combined with increased hyperinflation and lung function decline. The procedure was repeated similarly, this time treating the native right lower lobe with a total of 15 LVRCs (2 × 100 mm, 8 × 125 mm, and 5 × 150 mm) in 52 minutes as seen in Figure 6. The second treatment did not improve immediately, but after some time the pulmonary function values increased as seen in Figure 1 and the RV/TLC declined to below 58, which means the patient was no longer hyperinflated as seen in Figure 5. The 6-minute walking distance was unchanged above 300 m. Over time both FEV1 and especially FVC continued to decline, with a simultaneous incline in RV/TLC suggesting worsening of NLH. Since the native lung has received maximum treatment with LVRCs and other lung volume reduction modalities are not suitable in this setting, the patient is now being evaluated for a right-sided SLTx.

**Figure 6:**
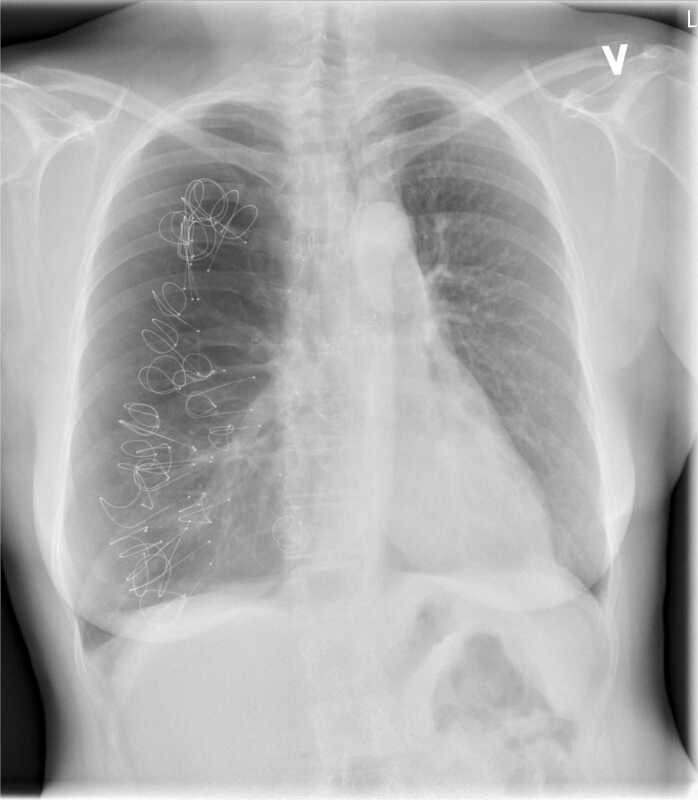
Chest X-ray after second LVRC treatment of right lower lobe. LVRC, lung volume reduction coils.

In conclusion, LVRC seems a safe treatment option when dealing with CV-positive NLH in SLTx recipients.

## Disclosure statement

All authors declare no financial disclosures related to this manuscript. There are no funding sources.

## Patient consent

Authors confirm that appropriate patient consent to publish this case report was received.
